# Supplementary material for: Content-rich biological network constructed by mining PubMed abstracts
Source: BMC Bioinformatics. 2004 Oct 8;5:147. doi: 10.1186/1471-2105-5-147 (PMC528731; doi:10.1186/1471-2105-5-147)
Supplement: Additional File 2 — The original results of the above study (non-essential files are deleted to keep the file size under the limit set by BMC bioinformatics). [file 1471-2105-5-147-S2.bz2 › chilibotAdditionalFile2/dip40/456ID10733106E10473/html/CCR3_CCL13.html]

 


 **CCR3** and **CCL13** 
  
Found 39 abstracts in PubMed, retrieved 39.  
 

 What does Google say? 
 PDF only 
| .edu only 

---

**Interactive relationship** (e.g. stimulation, inhibition, etc)

**Inhibitory relationship**- The compounds were potent inhibitors of eotaxin and MCP 4  [ **CCL13** ]  induced Ca 2 mobilization in RBL 2H3  **CCR3**  cells and eosinophils.  Ref: 10969084 J Biol Chem, 2000
- MCP 4  [ **CCL13** ]  inhibited binding of 125I eotaxin to eosinophils and  **CCR3**  transfected cells and inhibited 125I MCP 1 binding to CCR2b transfectants.  Ref: 9062350 J Clin Invest, 1997
- Preincubation of eosinophils with anti  **CCR3**  Ab 7B11 completely blocked eosinophil TEM induced by eotaxin, MCP 4  [ **CCL13** ] , and RANTES.  Ref: 10725746 J Immunol, 2000
- Identification of potent, selective non peptide CC chemokine receptor 3  [ **CCR3** ]  antagonist that inhibits eotaxin, eotaxin 2, and monocyte chemotactic protein 4  [ **CCL13** ]  induced eosinophil migration.  Ref: 10969084 J Biol Chem, 2000
- The  **CCR3**  blocking antibody also affected exocytosis it abrogated histamine and leukotriene release induced by eotaxin, and partially inhibited the response to RANTES and MCP 4  [ **CCL13** ] .  Ref: 9276730 J Clin Invest, 1997
- I TAC, Mig, and IP10 compete for the binding of eotaxin to  **CCR3**  bearing cells and inhibit migration and Ca 2 changes induced in such cells by stimulation with eotaxin, eotaxin 2, MCP 2 monocyte chemottractant protein 2, MCP 3, MCP 4  [ **CCL13** ] , and RANTES regulated on activation normal T cell expressed and secreted.  Ref: 11110785 J Biol Chem, 2001
**Neutral relationship**- Eotaxin 2 also displaced 125I eotaxin bound to the cloned  **CCR3**  stably expressed in CHO cells CHO  **CCR3**  and to freshly isolated human eosinophils with affinities similar to eotaxin and MCP 4  [ **CCL13** ] .  Ref: 9365122 J Leukoc Biol, 1997
- ... MCP 4  [ **CCL13** ]  exhibited a biphasic response consistent with sequential activation of  **CCR3**  at lower concentrations and CCR2 at 10 nM MCP 4  [ **CCL13** ]  and above.  Ref: 11120855 J Immunol, 2000
- Eosinophil leukocytes express high numbers of the chemokine receptor  **CCR3**  which binds eotaxin, monocyte chemotactic protein MCP 4  [ **CCL13** ] , and some other CC chemokines.  Ref: 9276730 J Clin Invest, 1997
- These data suggest that 1 eotaxin has a role in the early 6 h recruitment of eosinophils, while eotaxin 2 and MCP 4  [ **CCL13** ]  appear to be involved in later 24 h infiltration of these  **CCR3**  cells.  Ref: 10491000 J Immunol, 1999
- **CCR3** , CCR5 and CCR2 expressing leukocyte populations were identified at sites of MCP 4  [ **CCL13** ]  expression.  Ref: 11400154 J Pathol, 2001
- Therefore, interactions of eotaxin s, eotaxin CCL11, RANTES CCL5, and MCP 1 CCL2, MCP 2 CCL8, MCP 3 CCL7, MCP 4  [ **CCL13** ]   **CCL13**  with  **CCR3**  are responsible for the recruitment of basophils, eosinophils and mast cells.  Ref: 12009564 Mol Immunol, 2002
- CC chemokine receptor 3  [ **CCR3** ]  is also activated by other promiscuous ligands such as RANTES and monocyte chemotactic protein 4  [ **CCL13** ] .  Ref: 11286622 J Invest Dermatol, 2001
- Several CC chemokines including eotaxin CCL 11, eotaxin 2 CCL 24, RANTES CCL 5, and monocyte chemotactic protein 3 MCP 3, CCL 7 and 4 MCP 4  [ **CCL13** ] , CCL 13  [ **CCL13** ]  are potent eosinophil chemotactic and activating peptides acting through CC chemokine receptor 3  [ **CCR3** ]   **CCR3** .  Ref: 10969084 J Biol Chem, 2000
- The beta chemokine receptor  **CCR3** , which recognizes the ligands eotaxin, eotaxin 2, monocyte chemotactic protein 3, MCP4  [ **CCL13** ] , and RANTES, plays a central role in this process, and antagonists to this receptor could have potential therapeutic use in the treatment of allergy.  Ref: 10640766 J Immunol, 2000
- MCP 4  [ **CCL13** ]  induced a calcium flux in HEK 293 cells transfected with the monocyte selective MCP 1 receptor CCR 2B and the eosinophil selective eotaxin receptor  [ **CCR3** ]  CCR 3  [ **CCR3** ] , but not in the more widely expressed CCR 1 or CCR 5.  Ref: 8955214 J Immunol, 1996
- Human eosinophils express the eotaxin receptor  [ **CCR3** ] ,  **CCR3** , but respond to a variety of CC chemokines apart from eotaxin, including RANTES, monocyte chemotactic protein MCP 2, MCP 3, and MCP 4  [ **CCL13** ] .  Ref: 9005985 J Clin Invest, 1997
- Since selective eotaxin and non selective C C chemokines such as RANTES, MCP 3 and MCP 4  [ **CCL13** ]  all stimulate eosinophils via  **CCR3** , this receptor is potentially a prime therapeutic target in the spectrum of diseases involving eosinophil mediated tissue damage.  Ref: 9464841 Eur J Immunol, 1997
- Pretreatment of the basophils with a  **CCR3**  blocking antibody abrogated the migration induced by eotaxin, RANTES, and by low to optimal concentrations of MCP 4  [ **CCL13** ] , but decreased only minimally the response to MCP 3.  Ref: 9276730 J Clin Invest, 1997
- Furthermore,  **CCR3**  transfected human embryonic kidney cells labeled with Fura 2AM exhibited a rapid rise in intracellular free calcium after stimulation with eotaxin, eotaxin 2, or MCP 4  [ **CCL13** ] , but not with MDC.  Ref: 10069890 J Allergy Clin Immunol, 1999

**Non-interactive relationship** (e.g. studied together, co-existance, homology, etc.)

- Complete cross desensitization was observed between eotaxin 2, eotaxin and MCP 4  [ **CCL13** ]  confirming activation via  **CCR3** .  Ref: 9182688 J Exp Med, 1997
- In all individuals examined, including allergic and eosinophilic donors, > 95% of the response of eosinophils to eotaxin, RANTES, MCP 2, MCP 3, and MCP 4  [ **CCL13** ]  was shown to be mediated through  **CCR3** .  Ref: 9005985 J Clin Invest, 1997
- Nanomolar concentrations of UCB35625 were sufficient to inhibit eosinophil shape change responses to MIP 1alpha, MCP 4  [ **CCL13** ] , and eotaxin, while greater concentrations could inhibit the chemokine induced internalization of both CCR1 and  **CCR3** .  Ref: 10854442 J Biol Chem, 2000
- Stably transfected Cynomolgus  **CCR3**  bound human eotaxin CCL11 with similar kinetics Kd 240 pM and was responsive to human  **CCR3**  ligands eotaxin CCL11, eotaxin 2 CCL24, and MCP4  [ **CCL13** ]   **CCL13**  in Ca 2 mobilization and chemotaxis assays, thus provides a useful alternative species model system for the analysis of modulators of eotaxin  **CCR3**  induced signaling and activation.  Ref: 12421570 Cytokine, 2002
- The chemokine receptor  **CCR3**  and ligands monocyte chemoattractant protein MCP 3, MCP 4  [ **CCL13** ] , regulated upon activation normal T cell expressed and secreted RANTES and eotaxins I and II are of particular relevance for the recruitment and activation of eosinophils.  Ref: 11340325 Int Arch Allergy Immunol, 2001
- This study demonstrates that  **CCR3**  is the major receptor for eotaxin, RANTES, and MCP 4  [ **CCL13** ]  in human basophils, and suggests that basophils and eosinophils, which are the characteristic effector cells of allergic inflammation, depend largely on  **CCR3**  for migration towards different chemokines into inflamed tissues.  Ref: 9276730 J Clin Invest, 1997
- Eosinophil chemotactic chemokines eotaxin, eotaxin 2, RANTES, monocyte chemoattractant protein 3 MCP 3, and MCP 4  [ **CCL13** ] , and C C chemokine receptor 3  [ **CCR3** ]  expression in bronchial biopsies from atopic and nonatopic Intrinsic asthmatics.  Ref: 10570327 J Immunol, 1999
- Other human  **CCR3**  agonists, such as MCP 3, RANTES and MCP 4  [ **CCL13** ]  failed to increase dermal eosinophil peroxidase activity at doses up to 1 microg site 1 whereas the latter did produce a small effect at 3 microg site 1.  Ref: 10516663 Br J Pharmacol, 1999
- MCP 4  [ **CCL13** ]  activated cell migration via either CCR2b or  **CCR3**  in mouse lymphoma cells transfected with these chemokine receptors.  Ref: 9062350 J Clin Invest, 1997
- The eotaxin receptor  [ **CCR3** ]  is particularly attractive as a target as it is expressed in high numbers on eosinophils, but not other leukocytes, and appears to be the major detector of the eosinophil for eotaxin and other chemokines such as MCP 4  [ **CCL13** ] .  Ref: 9356998 Methods Enzymol, 1997
- These ligands include the  **CCR3**  selective chemokines eotaxin and eotaxin 2 and the more promiscuous CC chemokines, MCP 4  [ **CCL13** ] , MCP 3, MCP 2 and RANTES.  Ref: 11306952 Int Arch Allergy Immunol< MedlineTA>Int Arch Allergy Immunol, 1997
- Compared with AC and NC, the numbers of EG2 cells and the cells expressing mRNA for eotaxin, eotaxin 2, RANTES, MCP 3, MCP 4  [ **CCL13** ] , and  **CCR3**  were significantly increased in AA and NAA p <.  Ref: 10570327 J Immunol, 1999
- However, degranulation was induced only by  **CCR3**  ligands, including MCP 3, MCP 4  [ **CCL13** ] , RANTES, and eotaxin.  Ref: 10984371 J Allergy Clin Immunol, 2000
- We examined the expression of eotaxin, eotaxin 2, RANTES, monocyte chemoattractant protein 3 MCP 3, MCP 4  [ **CCL13** ] , and  **CCR3**  in the bronchial mucosa from atopic AA and nonatopic intrinsic.  Ref: 10570327 J Immunol, 1999
- Our data suggest that eotaxin, eotaxin 2, MCP 4  [ **CCL13** ] , and RANTES induce eosinophil TEM via  **CCR3**  with varied potency and efficacy.  Ref: 10725746 J Immunol, 2000
